# RDCRNet: RGB-T Object Detection Network Based on Cross-Modal Representation Model

**DOI:** 10.3390/e27040442

**Published:** 2025-04-19

**Authors:** Yubin Li, Weida Zhan, Yichun Jiang, Jinxin Guo

**Affiliations:** The College of Electronic and Information Engineering, Changchun University of Science and Technology, Changchun 130022, China; lyb_cust@163.com (Y.L.); jiangyichun@cust.edu.cn (Y.J.); guojinxin@cust.edu.cn (J.G.)

**Keywords:** object detection, multimodal, cross-modal representation, pretraining

## Abstract

RGB-thermal object detection harnesses complementary information from visible and thermal modalities to enhance detection robustness in challenging environments, particularly under low-light conditions. However, existing approaches suffer from limitations due to their heavy dependence on precisely registered data and insufficient handling of cross-modal distribution disparities. This paper presents RDCRNet, a novel framework incorporating a Cross-Modal Representation Model to effectively address these challenges. The proposed network features a Cross-Modal Feature Remapping Module that aligns modality distributions through statistical normalization and learnable correction parameters, significantly reducing feature discrepancies between modalities. A Cross-Modal Refinement and Interaction Module enables sophisticated bidirectional information exchange via trinity refinement for intra-modal context modeling and cross-attention mechanisms for unaligned feature fusion. Multiscale detection capability is enhanced through a Cross-Scale Feature Integration Module, improving detection performance across various object sizes. To overcome the inherent data scarcity in RGB-T detection, we introduce a self-supervised pretraining strategy that combines masked reconstruction with adversarial learning and semantic consistency loss, effectively leveraging both aligned and unaligned RGB-T samples. Extensive experiments demonstrate that RDCRNet achieves state-of-the-art performance on multiple benchmark datasets while maintaining high computational and storage efficiency, validating its superiority and practical effectiveness in real-world applications.

## 1. Introduction

Object detection, a critical branch of computer vision, has been extensively studied and is widely applied in fields such as autonomous driving [1,2], remote sensing [3,4], and military defense [5,6]. Typically, high-quality images captured by devices provide abundant target information. However, under low-light or adverse weather conditions, traditional RGB cameras often fail to capture sufficient effective information [7]. In contrast, thermal infrared imaging technology can function effectively in environments with insufficient lighting and is unaffected by common visual disturbances such as smoke and dust [8]. Consequently, researchers have increasingly explored multimodal perception technologies, combining visible light and thermal infrared sensors to develop RGB-T (RGB-thermal) sensing systems that overcome the limitations of single-modality systems.

Nevertheless, significant differences exist between the imaging mechanisms of RGB-T infrared images [9]. These differences manifest not only in the visual appearance of the images but also in how they fundamentally influence subsequent analysis and processing tasks [10,11]. Therefore, in object detection applications, effectively integrating RGB-T data and addressing the inherent structural and semantic disparities between the two modalities remains a major challenge in the research community. Since Ha et al. [12] pioneered the first end-to-end model MF-Net for RGB-T image object detection, substantial advancements have emerged in this domain [13,14,15]. Numerous state-of-the-art models have been innovated in network architecture design, introducing various efficient modal interaction mechanisms that significantly enhance the processing capabilities for RGB-T images [16,17,18]. However, these methods predominantly rely on precisely registered RGB-T infrared image datasets, restricting their applicability to more commonly available unaligned RGB-T devices.

In recent years, several researchers have addressed this challenge by proposing network architectures that adapt to slight misalignments between modalities, enabling effective interaction across different spatial and modal domains [19,20,21]. However, the scarcity of labeled RGB-T datasets often leads to overfitting during model training, resulting in suboptimal detection performance and limited generalization capabilities. Furthermore, current approaches frequently fail to fully account for the inherent distributional disparities between RGB and thermal infrared images during architectural design, leading to inadequate or inappropriate information fusion strategies that ultimately compromise the accuracy and robustness of detection systems.

To address the aforementioned challenges, we propose the RGB-T Object Detection Network Based on Cross-Modal Representation Model (RCDRNet), featuring a novel pipeline architecture and an innovative pretraining methodology. Specifically, we designed a Cross-Modal Representation Network (CRN) as the backbone, which initially encodes multimodal features independently through multilevel dense residual modules with shared parameters. These features then flow through a meticulously crafted Cross-Modal Aggregation Module (CAM) to enhance inter-modal information interaction. Within the CAM, we introduce Cross-modal Feature Remapping (CFR) and a Cross-Modal Refinement and Interaction Module (CRIM), which effectively address significant distribution disparities between cross-modal features and enable more efficient and robust information fusion.

Furthermore, we developed a cross-scale feature integration module that maximizes feature utilization efficiency by leveraging cross-modal features at multiple scales, thereby ensuring reliable multiscale prediction outputs. To strengthen the CRN’s feature extraction and cross-modal information fusion capabilities, we propose a cross-modal pretraining strategy that integrates masked reconstruction with semantic consistency loss functions. This approach adaptively learns semantic priors from unlabeled, unaligned RGB-T images. Beyond conventional reconstruction loss for pixel prediction, our method employs generative adversarial networks to capture implicit distributions from limited sample data.

## 2. Related Work

### 2.1. Traditional Object Detection Methods

In recent years, object detection has evolved from traditional machine learning methods to deep learning approaches. Early techniques relied on handcrafted features and classifiers like SVM [22] or AdaBoost [23], which, despite their effectiveness in specific scenarios, were computationally expensive and lacked generalization capabilities, rendering them inadequate for complex real-world environments.

The emergence of deep learning, particularly Convolutional Neural Networks (CNNs) [24], has revolutionized object detection, primarily categorized into two-stage and single-stage methods. A prominent two-stage method is R-CNN [25], which employs selective search to generate candidate regions and CNNs for feature extraction, substantially enhancing accuracy and efficiency. Fast R-CNN [26] and Faster R-CNN [27] further optimized the process by sharing feature extraction computations and introducing the Region Proposal Network (RPN), improving both speed and accuracy. More recent advances, such as the rotation-based framework by Xie et al. [28] and the sparse object detection by Sun et al. [29], have introduced sophisticated optimizations, including generating high-quality rotated boxes and eliminating the need for non-maximum suppression (NMS).

In the realm of single-stage methods, object detection is formulated as a regression problem that simultaneously handles bounding box prediction and classification. The YOLO family [30,31,32,33] directly predicts bounding boxes and class probabilities, achieving end-to-end detection with remarkable speed improvements. SSD [34] combines YOLO’s regression approach with Faster R-CNN’s anchor mechanism, enhancing small object detection by making predictions across multiple feature map scales. Subsequent innovations, such as FCOS [35], eliminated predefined anchor boxes in favor of direct bounding box regression from pixel points, while EfficientDet [36] introduced BiFPN (Bidirectional Feature Pyramid Network) for efficient multiscale feature fusion, striking an optimal balance between accuracy and computational efficiency. Cascade-DETR [37] enhanced Transformer-based detectors through cascade attention layers and an IoU prediction mechanism, significantly improving generalization capabilities and localization accuracy.

Despite these significant advancements, enhancing feature extraction alone cannot fully address the challenges posed by degraded image quality. Therefore, in the following sections, we will explore RGB-T object detection algorithms that leverage complementary thermal information.

### 2.2. RGB-T Object Detection Methods

In recent years, object detection has undergone a paradigm shift from conventional machine learning approaches to deep-learning-based methods. Early techniques predominantly relied on handcrafted features coupled with classifiers such as SVM [22] or AdaBoost [23]. While demonstrating efficacy in constrained scenarios, these methods suffered from computational inefficiency and limited generalization capacity, significantly restricting their applicability in complex real-world environments.

The advent of deep learning, particularly Convolutional Neural Networks (CNNs) [24], has fundamentally transformed the landscape of object detection, giving rise to two principal categories: two-stage and single-stage detection frameworks. Among two-stage methods, R-CNN [25] represents a seminal work that pioneered the integration of selective search for region proposal generation with CNN-based feature extraction, achieving substantial improvements in both accuracy and computational efficiency. Subsequent refinements including Fast R-CNN [26] and Faster R-CNN [27] introduced critical innovations such as shared convolutional features and the Region Proposal Network (RPN), further optimizing the speed–accuracy trade-off. Contemporary advancements exemplified by Xie et al.’s rotation-based framework [28] and Sun et al.’s sparse detection approach [29] have introduced sophisticated mechanisms for generating precise rotated bounding boxes and eliminating the computational overhead of non-maximum suppression (NMS).

Single-stage detectors formulate object detection as a unified regression problem encompassing simultaneous bounding box prediction and classification. The YOLO series [30,31,32,33] established a new paradigm through direct prediction of bounding coordinates and class probabilities, enabling real-time end-to-end detection. SSD [34] enhanced this framework by incorporating multiscale feature maps and anchor-based detection, significantly improving performance on small objects. Recent innovations such as FCOS [35] have further simplified the detection pipeline by eliminating anchor boxes entirely, while EfficientDet [36] introduced the BiFPN architecture for optimized multiscale feature fusion, achieving state-of-the-art efficiency. The Cascade-DETR framework [37] represents a significant advancement in Transformer-based detection, employing cascaded attention mechanisms and IoU-aware prediction to enhance both generalization capability and localization precision.

Despite these remarkable advancements, conventional approaches relying solely on improved feature extraction remain inadequate for addressing challenges posed by severe image quality degradation. This limitation motivates our subsequent investigation into RGB-T object detection methods that exploit complementary multimodal information.

## 3. Method

Current state-of-the-art RGB-T object detection models predominantly adopt end-to-end architectures to streamline both training and inference procedures. While this design paradigm offers operational convenience, it exhibits strong dependence on large-scale training datasets with precise manual annotations. Such heavy reliance on annotated data imposes significant constraints on further advancements in this field. Particularly in RGB-T multimodal fusion scenarios, the inherent data scarcity exacerbates these challenges. Theoretically, transfer learning could alleviate this data insufficiency by introducing relevant prior knowledge through pretraining on datasets from related tasks. However, the application of transfer learning in RGB-T object detection remains relatively unexplored, primarily due to the absence of large-scale annotated thermal infrared datasets. Moreover, establishing effective cross-modal prior knowledge constitutes a critical research challenge that demands immediate attention.

**Figure 1:**
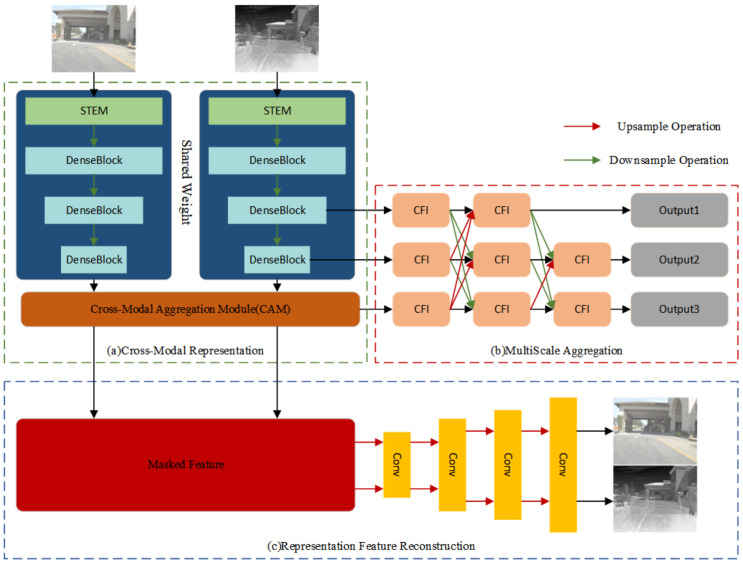
The overall network architecture of RDCRNet.

To effectively exploit complementary information in RGB-T data and adaptively construct semantic representations from unlabeled samples, we propose RDCRNet—a novel network architecture that enhances model performance through representation–interpretation decoupling and differentiated training strategies. Figure 1 illustrates RDCRNet’s design and inference pipeline. The architecture comprises three key components: (1) a Cross-Modal Representation Network serving as the backbone, (2) a multiscale aggregation module for inference, and (3) a Representation Feature Reconstruction mechanism for training. Through coordinated operation enabled by joint training strategies, these components collectively capture implicit prior knowledge from RGB-T data, substantially improving the detection system’s overall performance and robustness. Subsequent sections detail these components’ mechanisms, functionalities, and specific training methods.

### 3.1. Cross-Modal Representation Network

As depicted in Figure 1a, the Cross-Modal Representation Network (CRN) consists of two principal elements: a feature extractor and the Cross-Modal Attention Module (CAM). The feature extractor is designed to derive high-level features from individual modalities, while CAM facilitates inter-modal information transfer and fusion. Although Transformer architectures demonstrate superior performance, they typically require extensive training data to effectively capture local image characteristics—a requirement impractical for RGB-T applications given dataset limitations. To address this, we implement a hybrid architecture combining CNNs and Transformers in the feature extractor and CAM, respectively, ensuring adequate contextual relationship modeling while preserving local inductive biases.

The CRN processes RGB images $I_{RGB}\in R^{H\times W\times 3}$ and thermal infrared images $I_{T}\in R^{H\times W\times 1}$, where the single-channel thermal input is replicated across three channels to maintain architectural consistency. Feature extraction initiates with an STEM module that projects input images into a high-dimensional latent space, generating shallow features $f_{RGB,0}\in R^{H/2\times W/2\times C_{0}}$ and $f_{T,0}\in R^{H/2\times W/2\times C_{0}}$ through the transformation:

(1) $$\begin{array}{c} f_{T,0}=STEM\left(I_{T}\right) \end{array}$$

(2) $$\begin{array}{c} f_{RGB,0}=STEM\left(I_{RGB}\right) \end{array}$$

where the parameter-shared STEM(·) operator performs simultaneous preliminary feature extraction and spatial downsampling for both modalities.

Following the design of most backbone networks, the entire network is divided into three stages, each processing RGB-T image features at different scales. To improve feature utilization and enhance representational capacity, we incorporate dense residual modules at each stage. Given the high dimensionality of infrared and visible light image data, which can lead to substantial computational and memory overhead, we integrate downsampling layers at multiple stages. These layers reduce the data dimensionality while enlarging the receptive field, enabling the model to output features at varying scales and depths. The features produced at each stage of the two branches are denoted as $f_{RGB,i}\in R^{H_{i}\times W_{i}\times C_{i}}$ and $f_{T,i}\in R^{H_{i}\times W_{i}\times C_{i}}$ (where i=1,2,3), with feature resolutions progressively reduced to 1/4, 1/8, and 1/16 of the original image size. Concurrently, the channel dimensions increase to 128, 256, 512, and 1024, ensuring sufficient information density in the extracted features. This operation can be represented as follows:

(3) $$\begin{array}{cc} \; & f_{RGB,i}=F_{i}\left(f_{RGB,i-1}\right) \end{array}$$

(4) $$\begin{array}{cc} \; & f_{T,i}=F_{i}\left(f_{T,i-1}\right) \end{array}$$

where parameter-shared $F_{i}\left(\cdot \right)$ represents the function of the dense residual block at the *i*-th stage. At the final stage of the network, the RGB and thermal infrared features $f_{RGB,3}\in R^{H_{3}\times W_{3}\times C_{3}}$ and $f_{T,3}\in R^{H_{3}\times W_{3}\times C_{3}}$ are embedded and transformed into feature vector sets $v_{RGB,i}\in R^{n\times C_{3}}$ and $v_{T,i}\in R^{n\times C_{3}}$. These feature vectors are subsequently processed through the CAM for feature refinement. By normalizing and integrating the cross-modal information, the transformed feature vector sets ${\overset{⌢}{v}}_{RGB,i}\in R^{n\times C}$ and ${\overset{⌢}{v}}_{T,i}\in R^{n\times C}$ are derived. For downstream processing, we restructure these feature vector collections back into feature maps, yielding $f_{RGB,out}\in R^{H_{3}\times W_{3}\times C}$ and $f_{T,out}\in R^{H_{3}\times W_{3}\times C}$. The inference and training phases employ distinct processing pipelines, with detailed procedures discussed in subsequent sections.

### 3.2. Cross-Modal Aggregation Module

**Figure 2:**
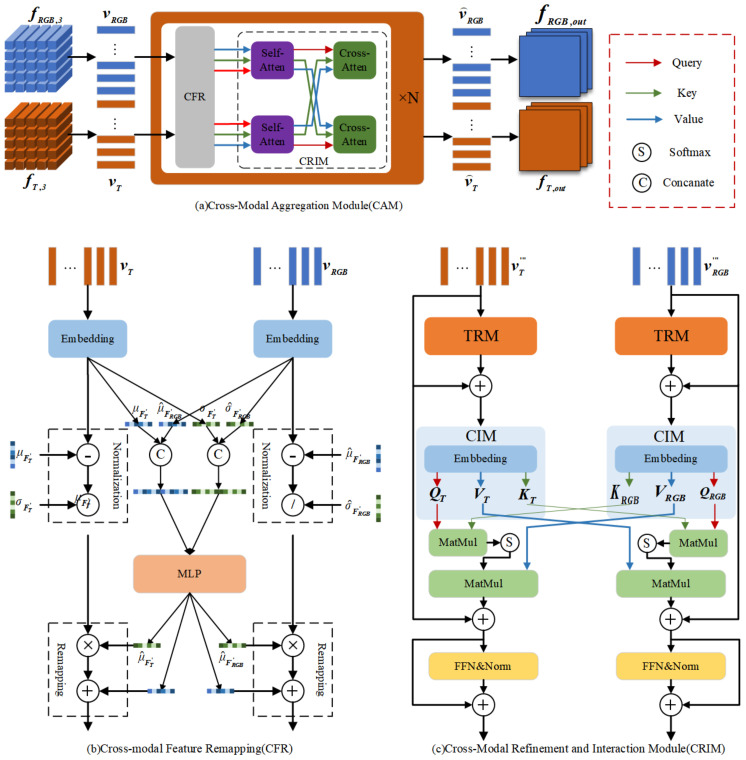
The structure of the Cross-Modal Aggregation Module.

In existing RGB-T object detection models, information interaction between different modality features is typically achieved through feature addition or concatenation, followed by refinement using learnable layers. While this approach is simple and effective, it struggles when processing non-registered RGB-T images, and lacks a standardized methodology. To address these limitations, we propose the Cross-Modal Aggregation Module within the CRN, which aims to overcome these shortcomings. As illustrated in Figure 2, the CAM comprises a series of stacked components, with the primary function of mitigating modality discrepancies and enhancing RGB-T feature fusion. The main functional components of the CAM include two parts: cross-modal feature remapping and information aggregation.

**Cross-Modal Feature Remapping (CFR).** In the CRN, we employ feature extractors with shared parameters to process RGB-T images, effectively reducing the network’s spatial complexity. However, due to the inherent differences in the imaging mechanisms of RGB and thermal infrared modalities, models without adaptive capabilities are susceptible to distributional discrepancies between these modalities during feature extraction, potentially degrading model performance significantly. To address this issue, we propose a Cross-Modal Feature Remapping Module, which adaptively recalibrates RGB-T features based on their statistical distributions. This remapping process helps mitigate modality discrepancies, ensuring more consistent feature representations for subsequent cross-modal information fusion.

The detailed architecture of the CFR module is depicted in Figure 4. Initially, after passing through an embedding layer, the extracted RGB and thermal infrared features are obtained, which can be mathematically represented as follows:

(5) $$\begin{array}{c} {v^{\prime }}_{RGB}=W_{1}v_{RGB}+b_{1}, \end{array}$$

(6) $$\begin{array}{c} {v^{\prime }}_{T}=W_{2}v_{T}+b_{2}, \end{array}$$

where *W* represents the weight parameters of the embedding layer, and *b* is the bias term. Inspired by prior research, modality differences are predominantly manifested in the statistical distribution properties of feature vectors, with mean and variance being the most characteristic parameters. To mitigate such modality discrepancies, we apply an initial normalization process to the extracted features, ensuring that feature vectors from both modalities conform to a standard normal distribution with zero mean and unit variance. This operation can be represented as follows:

(7) $$\begin{array}{c} {v^{\prime \prime }}_{RGB}=\frac{{v^{\prime }}_{RGB}-\mu_{{v^{\prime }}_{RGB}}}{\sigma_{{v^{\prime }}_{RGB}}} \end{array}$$

(8) $$\begin{array}{c} {v^{\prime \prime }}_{T}=\frac{{v^{\prime }}_{T}-\mu_{{v^{\prime }}_{T}}}{\sigma_{{v^{\prime }}_{T}}} \end{array}$$

where $\left[\mu_{{v^{\prime }}_{RGB}},\mu_{{v^{\prime }}_{T}}\right]$ and $\left[\sigma_{{v^{\prime }}_{RGB}},\sigma_{{v^{\prime }}_{T}}\right]$ represent the mean and variance of the feature vectors ${v^{\prime \prime }}_{RGB}$ and ${v^{\prime \prime }}_{T}$, respectively. The aforementioned normalization method processes each feature vector independently. However, in practical applications, target objects are typically represented by multiple feature vectors. Therefore, we preserve the mean and variance information of the RGB and thermal infrared feature vectors across different channels and concatenate these statistics into a composite feature vector. Subsequently, through a two-layer multilayer perceptron (MLP), we correlate these key statistics and adaptively generate correction parameters $\left[\mu_{RGB},\mu_{T}\right]$ and $\left[\sigma_{RGB},\sigma_{T}\right]$ based on the global cross-modal information. This operation can be represented as follows:

(9) $$\begin{array}{c} \left[\mu_{RGB},\mu_{T}\right]=f_{MLP}^{1}\left(\left[\mu_{{v^{\prime }}_{RGB}},\mu_{{v^{\prime }}_{T}}\right]\right) \end{array}$$

(10) $$\begin{array}{c} {}[\sigma_{RGB},\sigma_{T}]=f_{MLP}^{2}\left(\left[\sigma_{{v^{\prime }}_{RGB}},\sigma_{{v^{\prime }}_{T}}\right]\right) \end{array}$$

where $f_{MLP}\left(\cdot \right)$ represents the equivalent function of the MLP. Finally, we perform the remapping operation on the normalized features using the correction parameters to obtain the output dual-band features ${v^{\prime \prime \prime }}_{RGB}$ and ${v^{\prime \prime \prime }}_{T}$. This operation can be expressed as follows:

(11) $$\begin{array}{c} {v^{\prime \prime \prime }}_{RGB}={v^{\prime }}_{RGB}\cdot \sigma_{RGB}+\mu_{RGB} \end{array}$$

(12) $$\begin{array}{c} {v^{\prime \prime \prime }}_{T}={v^{\prime }}_{T}\cdot \sigma_{T}+\mu_{T} \end{array}$$

**Cross-Modal Refinement and Interaction Module(CRIM).** After completing the RGB-T feature remapping, a critical challenge emerges: determining how to effectively process and fuse these cross-modal features. To address this, we propose the Cross-Modal Refinement and Interaction Module (CRIM), whose detailed architecture is illustrated in Figure 2c. CRIM comprises three core components: the Trinity Refinement Module (TRM), the Cross-Attention Interaction Module (CIM), and the Feed-Forward Network (FFN).

**Figure 3:**
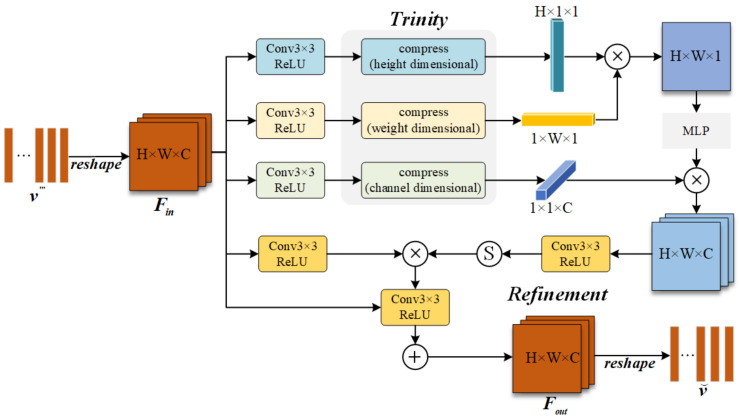
The structure of the Trinity Refinement Module.

For both modalities, we first employ a shared-parameter encoder for preliminary feature processing. To further enhance intra-modal and inter-modal information extraction, we designed the Trinity Refinement Module, which refines feature granularity while strengthening both contextual information and inter-channel relationships. The architecture of TRM is detailed in Figure 3.

The vector $v^{\prime \prime \prime }$ is reshaped into an input feature map $F_{in}$, which undergoes dimensional compression to capture global contextual information. Through two matrix multiplication operations, the information extracted from three dimensions is aggregated to obtain a robust global feature representation. This process effectively captures long-range dependencies, significantly enhancing the modal features’ expressive power. The integrated global information serves as pixel-level attention weights, applied to input features via element-wise multiplication for key information extraction.

To mitigate information loss and gradient vanishing, we incorporate local skip connections. The final output feature map $F_{out}$ is converted back to vector form $\overset{⌣}{v}$ for subsequent processing. These operations are formulated as follows:

(13) $$\begin{array}{c} \begin{array}{c} F_{d}=f_{conpress,d}\left(f_{conv}\left(F_{in}\right)\right),\forall d\in \left(H,W,C\right) \end{array} \end{array}$$

(14) $$\begin{array}{c} \begin{array}{c} F_{G}=f_{conv}\left(f_{MLP}\left(F_{H}F_{W}\right)F_{C}\right) \end{array} \end{array}$$

(15) $$\begin{array}{c} F_{out}=f_{conv}\left(F_{G}\cdot f_{conv}\left(F_{in}\right)\right)+F_{in} \end{array}$$

where $f_{\mathrm{compress},d}\left(\cdot \right)$ denotes the dimension-wise compression operation employing adaptive average pooling across height, width, and channel dimensions. While maintaining robust detection performance for unaligned RGB-T images remains our objective, the aforementioned operations alone prove insufficient for facilitating effective cross-modal interaction between RGB and thermal modalities.

Existing approaches predominantly focus on aligned RGB-T image pairs, lacking the capability for feature matching under spatial misalignment. To address this limitation, we propose a Cross-Modal Self-Attention Mechanism that enables comprehensive feature matching and fusion between misaligned RGB-T modalities. The mechanism operates as follows:

(16) $$\begin{array}{c} \begin{array}{ccc} Q_{RGB}=W_{Q}{\overset{⌣}{v}}_{RGB} & K_{RGB}=W_{K}{\overset{⌣}{v}}_{RGB} & V_{RGB}=W_{V}{\overset{⌣}{v}}_{RGB} \end{array} \end{array}$$

(17) $$\begin{array}{c} \begin{array}{ccc} Q_{T}=W_{Q}{\overset{⌣}{v}}_{T} & K_{T}=W_{K}{\overset{⌣}{v}}_{T} & V_{T}=W_{V}{\overset{⌣}{v}}_{T} \end{array} \end{array}$$

(18) $$\begin{array}{c} {\tilde{v}}_{T}=\mathrm{Softmax}(\frac{Q_{T}\cdot {K_{RGB}}^{T}}{\sqrt{d_{k}}})V_{\mathrm{RGB}}+{v^{\prime \prime \prime }}_{T} \end{array}$$

(19) $$\begin{array}{c} {\tilde{v}}_{RGB}=\mathrm{Softmax}(\frac{Q_{RGB}\cdot {K_{T}}^{T}}{\sqrt{d_{k}}})V_{T}+{v^{\prime \prime \prime }}_{RGB} \end{array}$$

where ${\overset{⌣}{v}}_{RGB}$ and ${\overset{⌣}{v}}_{T}$ represent the input feature vectors from the RGB and T modalities, respectively; $Q_{RGB}$, $K_{RGB}$, and $V_{RGB}$ represent the query, key, and value vectors derived from ${\overset{⌣}{v}}_{RGB}$; $W_{Q}$, $W_{K}$, and $W_{V}$ are learnable linear transformation matrices. Similarly, $Q_{T}$, $K_{T}$, and $V_{T}$ denote the query, key, and value vectors obtained from ${\overset{⌣}{v}}_{T}$. $d_{k}$ represents the feature dimension for each attention head, and $\sqrt{d_{k}}$ serves as a scaling factor to prevent excessively large values in the dot product. The cross-modal attention computes similarity scores between the RGB key $K_{RGB}$ and the TIR query $Q_{T}$ via dot product, followed by normalization with factor $\sqrt{d_{k}}$ to obtain attention weights. These weights are then applied to perform a weighted summation of the RGB value $V_{RGB}$ to update the TIR features, denoted as ${\tilde{v}}_{T}$. Similarly, RGB features are updated via the TIR key and value to obtain ${\tilde{v}}_{RGB}$. $v_{T}^{\prime \prime \prime }$ and $v_{RGB}^{\prime \prime \prime }$ represent residual connections that preserve original feature information.

In the RGB-T cross-attention mechanism, the query vector $Q_{T}$ from the TIR modality interacts with the key vector $K_{RGB}$ from the RGB modality to compute similarity scores through dot product operations. These scores are subsequently normalized using a softmax function to generate attention weights. The weighted summation of RGB value vectors $V_{RGB}$ is then computed to update the TIR features. Conversely, the query vector $Q_{RGB}$ interacts with the TIR key $K_{T}$, and the weighted summation of $V_{T}$ updates the RGB features. This bidirectional cross-modal attention facilitates effective feature matching and fusion between RGB and TIR modalities, ensuring robust feature alignment even under conditions of spatial misalignment.

To further enhance the representational capacity of the model, we incorporate an FFN at the end of the CRIM module. By introducing additional non-linear transformations, the FFN significantly improves the model’s performance. This operation can be mathematically expressed as follows:

(20) $$\overset{⌢}{v}=\mathrm{LayerNorm}\left(f_{FFN}\left(\tilde{v}\right)\right)+\tilde{v}$$

where LayerNorm(·) denotes the layer normalization function, and $f_{FFN}\left(\cdot \right)$ represents the function implemented by the Feed-Forward Neural Network (FFN).

### 3.3. Cross-Modal Representation PreTraining

**Figure 4:**
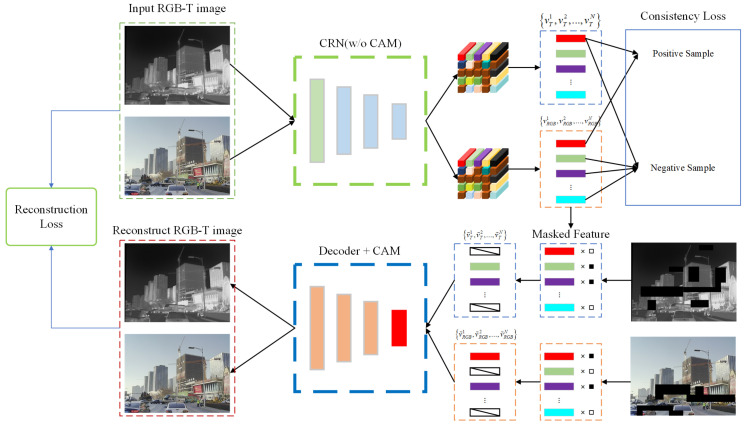
A schematic diagram of cross-modal representation pretraining.

Although the CRN we designed exhibits strong potential for information complementarity, its performance heavily depends on the implementation of appropriate training methodologies. In existing RGB-T datasets, the quantity of registered and annotated samples is substantially smaller than that of unaligned and unannotated ones. To address this imbalance, we aim to develop an effective approach that can extract useful prior knowledge from a broader spectrum of datasets. Our proposed pretraining method, as illustrated in Figure 4, comprises two primary steps, in addition to training the CRN on the conventional ImageNet and COCO datasets to provide it with foundational capabilities.

Inspired by Masked Autoencoders (MAEs) [38,39], we propose a cross-modal masked reconstruction method. This approach facilitates collaborative learning of cross-modal features by strategically applying partial masking to the encoded features of both modalities. Regarding mask generation, unlike traditional MAEs, we introduce additional constraints during the generation process to ensure that critical information required for reconstruction must simultaneously originate from both the source modality and the reference modality. This significantly enhances the model’s feature extraction capabilities and the complementary information between modalities. To achieve this, we impose constraints on the minimum and maximum overlap rates during mask generation. This unique design ensures a balanced information sharing between source and reference modalities: on one hand, it effectively prevents the model from over-relying on information from a single modality, encouraging full exploration and leveraging of each modality’s unique advantages; on the other hand, it significantly enhances the model’s sensitivity and adaptability to cross-modal information, thereby establishing a more robust foundation for subsequent downstream tasks.

During the pretraining stage, we refined and optimized the CRN structure to more efficiently extract and integrate multimodal information. Specifically, in the encoding component of the CRN, we removed certain elements of the CAM that focus exclusively on image feature encoding, and applied masking to the features. After feature encoding is completed, the masked encoded features are transmitted to the decoder, which incorporates the CAM, for reconstruction. The CAM serves a pivotal function in this process: it dynamically captures semantic correlations between source and reference modalities, excavates and leverages the complementary characteristics of both modalities, and achieves efficient cross-modal information fusion. This architecture requires the decoder not only to restore masked image information but also to fully utilize cross-modal synergy for precise comprehensive modeling of the overall content. This integration of modality interaction with preservation of image self-information enables the model to demonstrate enhanced cross-modal understanding capabilities in the reconstruction task while improving self-supervised learning effectiveness.

We employ the root mean square error (RMSE) as the primary reconstruction loss:

(21) $$l_{rec}=\lambda_{1}\cdot \frac{1}{p_{RGB}}∥I_{RGB}-{I^{\prime }}_{RGB}∥+\lambda_{2}\cdot \frac{1}{p_{T}}∥I_{T}-{I^{\prime }}_{T}∥$$

where ${I^{\prime }}_{RGB}$ and ${I^{\prime }}_{T}$ represent the reconstructed RGB and thermal infrared images, and $p_{RGB}$ and $p_{T}$ denote the total number of pixels in the RGB and infrared images. However, due to the limited scale of collected RGB-T samples, which represent only a restricted subset of real-world scenarios, relying solely on MSE may neglect semantic information and complex patterns in the data. Therefore, we incorporate Generative Adversarial Learning (GAN) [40,41] to learn the implicit data distribution and capture richer patterns, which is validated in subsequent experiments. Beyond adversarial loss, we also introduce perceptual loss to ensure semantic consistency between reconstructed and real images. The expression for perceptual loss is as follows:

(22) $$\begin{array}{c} l_{adv}={∥1-D(G\left({I^{\prime }}_{RGB},{I^{\prime }}_{T}\right))∥}^{2} \end{array}$$

(23) $$\begin{array}{c} l_{per}=\sum_{j\in \Omega }{∥\phi_{j}\left(G\left({I^{\prime }}_{RGB},{I^{\prime }}_{T}\right)\right)-\phi_{j}\left({I^{\prime }}_{RGB},{I^{\prime }}_{T}\right)∥}^{2} \end{array}$$

where G(·) denotes the CRN functioning as the generator, D(·) represents the discriminator model, and $\varphi_{j}\left(\cdot \right)$ indicates the features extracted from the *j*-th layer of the VGG network. Although unaligned samples significantly outnumber registered samples, the registered samples spatially align the semantic and texture information of RGB-T modalities, thereby providing more robust supervisory signals.

To ensure semantic consistency during the reconstruction process of registered RGB-T images, we incorporate a contrastive loss [42,43] to aggregate semantic information from corresponding regions across the two modalities. Specifically, we select N sets of feature vectors from different spatial locations, designating feature vectors from identical locations as positive samples and optimizing them to be maximally proximate in the feature space during training. Conversely, feature vectors from different locations are designated as negative samples and are optimized to be maximally distant in the feature space. The expression for this contrastive loss function is as follows:

(24) $$l_{ct}=-\log\frac{\exp(sim\left(v_{RGB,i},v_{T,i}\right)/\tau )}{\sum_{1}^{N}1_{\left[i\neq j\right]}\exp\left(sim\left(v_{RGB,i},v_{T,j}\right)/\tau \right)}$$

where sim(·,·) denotes the similarity measurement function, and the cosine similarity $sim\left(x,y\right)=x^{T}y/\left(∥x∥\cdot ∥y∥\right)$ is employed in this work. Ultimately, we formulate a hybrid loss function that integrates the aforementioned losses to govern the CRN training process. The expression for this comprehensive loss is as follows:

(25) $$l=l_{rec}+l_{adv}+l_{per}+s\left(I_{RGB},I_{T}\right)\cdot l_{ct}$$

where s(·,·) represents the indicator function that outputs 0 for unaligned samples and 1 for aligned samples. Through this methodology, we can effectively extract latent information from a substantial volume of unaligned RGB-T images while simultaneously leveraging the semantic and texture information embedded in registered samples for effective supervision.

### 3.4. Object Detection

After extracting the requisite features through the CRN, the subsequent task involves decoding these abstract representations into precise target localization and dimensional information. This decoding process is implemented via a meticulously designed decoder architecture, which is elaborated in the following sections.

**Figure 5:**
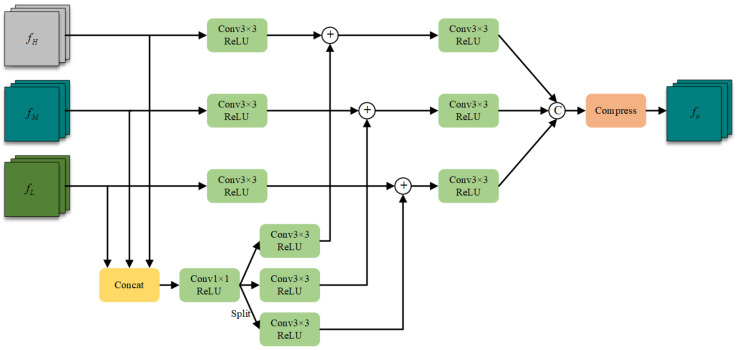
The structure of the Cross-Scale Feature Integration Module.

**Cross-Scale Feature Integration.** Numerous research efforts have demonstrated that enhancing the processing and aggregation of multiscale features constitutes a pivotal strategy for addressing the challenge of target scale variation. Building upon this established approach, we introduce the Cross-Scale Feature Integration Module (CFI), which performs dense fusion of features across various scales and generates three feature maps at different resolutions. The architectural framework of the CFI is illustrated in Figure 5.

The feature maps processed by the output head ultimately encode the positional coordinates, dimensional parameters, and categorical attributes of targets at multiple scales. Through this architectural design, we ensure comprehensive information exchange across different scales, substantially enhancing the model’s capacity to detect targets of varying dimensions and improving overall detection accuracy and robustness across diverse operational scenarios.

**Loss Function.** In object detection tasks, precise localization loss is indispensable. Therefore, we adopt the loss function from the YOLOv8 model [33], which has demonstrated excellent performance. The total loss function is defined as follows:

(26) $$l=l_{VFL}+l_{CIoU}+l_{DFL}$$

where $l_{VFL}$ represents the classification loss and $l_{CIoU}+l_{DFL}$ constitute the regression loss. The expressions for these components are

(27) $$\begin{array}{cc} \; & l_{VFL}=\frac{1}{N}\sum_{i=1}^{N}\left(q_{i}\cdot \log\left(p_{i}\right)+\left(1-q_{i}\right)\cdot \log\left(1-p_{i}\right)\right) \end{array}$$

(28) $$\begin{array}{cc} \; & l_{CIoU}=1-IoU+\frac{\rho^{2}\left(b,b^{gt}\right)}{c^{2}}+\alpha v \end{array}$$

where *N* denotes the total number of samples, $q_{i}$ represents the ground truth label of sample *i* (1 for positive samples and 0 for negative samples), and $p_{i}$ indicates the predicted probability of sample *i*. In the CIoU loss, IoU measures the intersection over union between the predicted bounding box and the ground truth box, $\frac{\rho^{2}\left(b,b^{gt}\right)}{c^{2}}$ quantifies the normalized Euclidean distance between the centers of the predicted box *b* and ground truth box $b^{gt}$, *c* represents the diagonal length of the smallest enclosing box covering both boxes, *v* measures the consistency of aspect ratios between the two boxes, and α serves as a weighting coefficient.

## 4. Experiment

### 4.1. Experimental Setup

**Figure 6:**
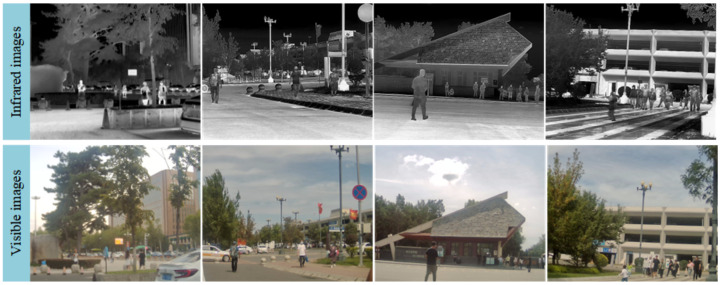
Examples of collected unaligned and unlabeled RGB-T image samples.

**Datasets and Metrics.** During the pretraining phase of CRN, we utilized aligned datasets MFNet [12] and KAIST [44], which contain 1569 and 15,000 infrared-visible image pairs, respectively. Additionally, the unaligned FLIR ADAS dataset, comprising a total of 31,569 image samples, was incorporated. To further enhance CRN’s training performance, we also collected a large number of unaligned and unlabeled RGB-T image samples, totaling 4200 images, some examples of which are shown in Figure 6. For the object detection task, we used the KAIST and FLIR ADAS datasets during both the training and testing phases, providing precise annotations for vehicles, pedestrians, and various road targets. The datasets were split into training, validation, and test sets in a 6:2:2 ratio. Notably, in the experiments involving the FLIR ADAS dataset, infrared images were used as the primary input data for object detection.

To comprehensively evaluate model performance, we employed standard detection metrics including accuracy, recall, precision, F1 score, mean intersection over union (mIoU), and mean average precision (mAP) to quantitatively assess the effectiveness of both multimodal fusion and object detection capabilities.

**Implementation Details.** All experiments were conducted on a high-performance computing platform featuring an Intel(R) Core™ i9-13900KF processor, 64 GB RAM, and an NVIDIA GeForce RTX 4090 GPU. The software environment consisted of the Windows 11 operating system with PyCharm 2021.3.2 as the integrated development environment and PyTorch 2.1.0 as the deep learning framework.

For the Cross-Modal Representation Network pretraining phase, we employed the ADAM optimizer with parameters $\beta_{1}=0.9$, $\beta_{2}=0.999$, and $\epsilon =8\times 10^{-8}$ to ensure stable convergence. We configured the training with a minibatch size of 16 and an initial learning rate of $5\times 10^{-4}$. The network was trained for 400 epochs, with learning rate decay to 10% of its current value implemented at the 200th and 300th epochs. Regarding the masking strategy, we set the masking rate at 0.6, with maximum and minimum overlaps for RGB-T masks configured at 0.5 and 0.2, respectively.

During the object detection training phase, we maintained the ADAM optimizer with identical parameter settings ($\beta_{1}=0.9$, $\beta_{2}=0.999$, and $\epsilon =8\times 10^{-8}$). We increased the minibatch size to 64 and utilized an initial learning rate of $1\times 10^{-2}$. The training process spanned 200 epochs, with learning rate reductions to 10% of the current value scheduled at the 100th and 150th epochs.

### 4.2. Comparison with SOTA Methods

As our approach optimizes the network structure and pretraining methods for unaligned data, it is applicable to both aligned and unaligned object detection. We conducted comparative experiments on both scenarios.

**Aligned Datasets.** For aligned RGB-T datasets, we compared our method with state-of-the-art RGB-T object detection approaches, including IAF R-CNN [45], Midway Fusion [46], CIAN [47], AR-CNN [48], LRAF-Net [49], CAGTDet [50], and CMX [14]. Additionally, we evaluated single-modal object detection methods such as Faster R-CNN and RetinaNet. All methods were trained on the same dataset using the experimental setups provided by their respective authors. The quantitative results are presented in Table 1. These results demonstrate that RGB-T object detection methods consistently outperform single-modal approaches, with the integration of cross-modal information significantly enhancing detection accuracy. Despite the varying architectures and fusion strategies employed by the RGB-T object detection networks used for comparison, the performance improvements across existing methods remain relatively modest. Among all the RGB-T detection methods, our approach achieved the best performance, with improvements in mIoU, F1 score, and mAP of 5.3%, 1.7%, and 3.5%, respectively, over the second-best method. This success can be attributed to our proposed cross-modal information interaction structure and effective pretraining strategy.

**Figure 7:**
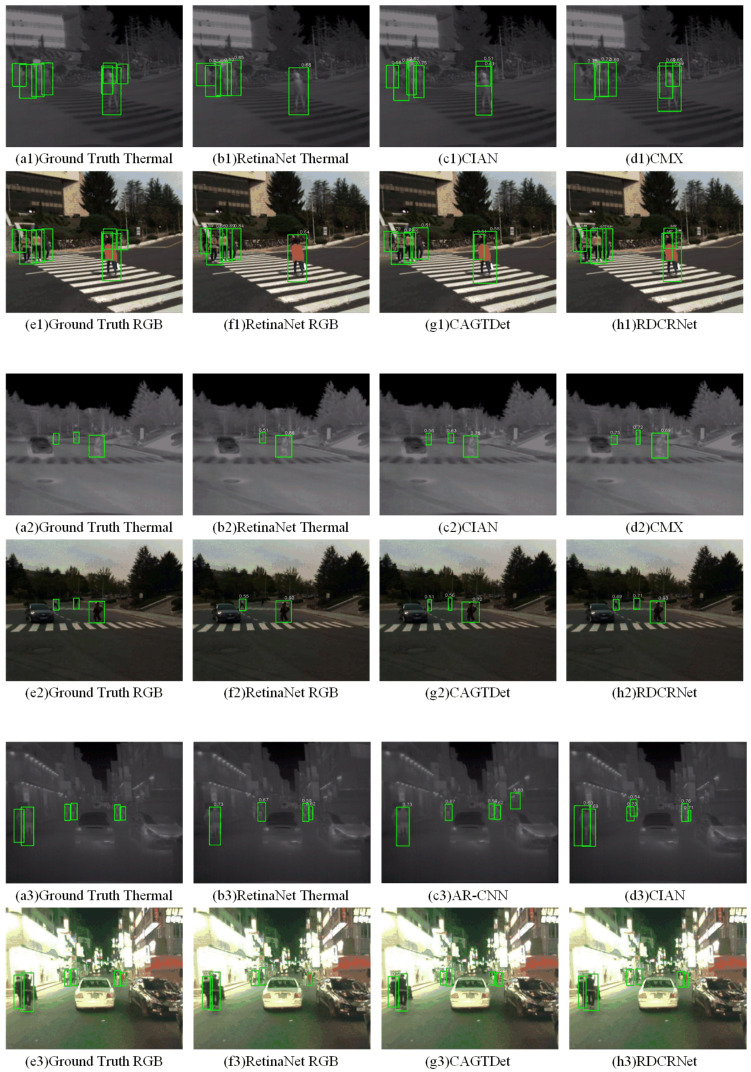
Qualitative comparison of various algorithms under the registration dataset (KAIST).

To provide a visual demonstration, several detection examples are presented in Figure 7. Detection using only infrared or visible modalities often resulted in missed or false detections due to insufficient brightness or contrast, respectively. Consistent with the quantitative evaluation metrics, RGB-T detection methods produced superior results. Among all the methods, ours accurately identified targets, achieved precise object boundaries, and demonstrated high-confidence responses. The qualitative and quantitative analyses are in strong agreement.

**Unaligned Datasets.** Unaligned RGB-T data are more prevalent in real-world applications, but they present significant challenges for algorithms. We conducted comparative experiments on unaligned datasets to evaluate our algorithm’s capability in addressing the issue of spatial misalignment in RGB-T data. Most existing methods struggle to handle unaligned data effectively due to the absence of structural design and training strategies tailored to matching disparate spatial features. As a result, we selected AR-CNN, LRAF-Net, and CAGTDet as comparative methods, as they can tolerate a certain degree of misalignment. To ensure that these methods performed optimally, we applied a rough alignment to the images from the FLIR dataset, mitigating excessive scale and spatial discrepancies. Additionally, we included several single-modal object detection methods, such as Faster R-CNN, RetinaNet, EfficientDet, and Cascade-DETR, which were trained and tested on infrared images, serving as baselines.

**Figure 8:**
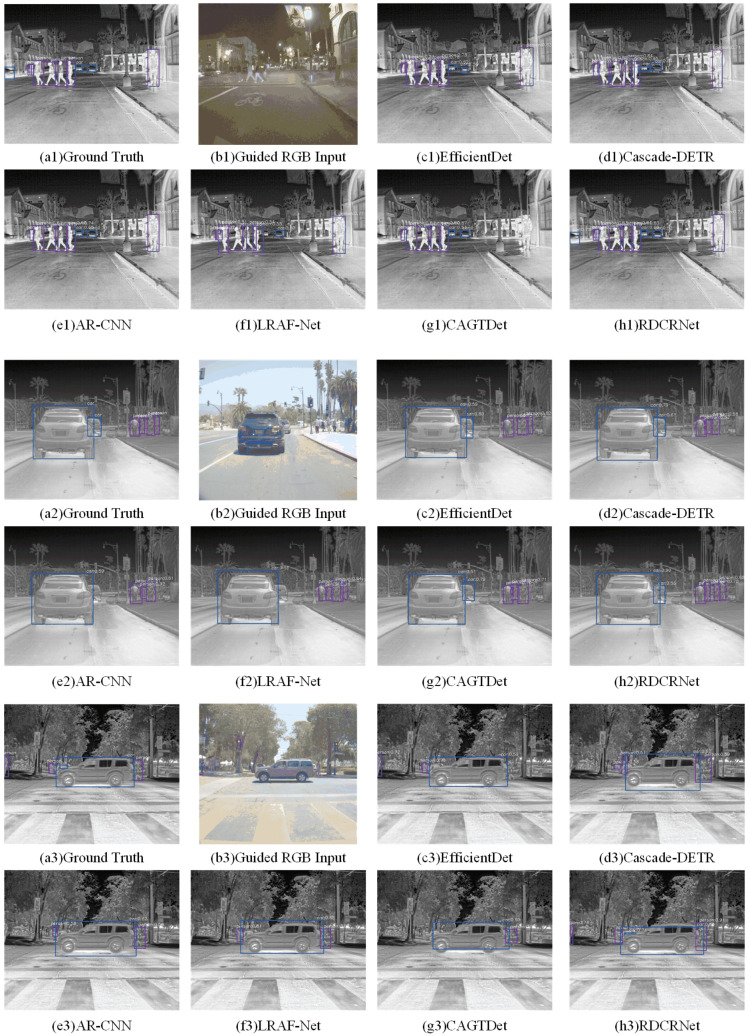
Qualitative comparison of various algorithms under the unregistration dataset (FLIR ADAS).

The results of the quantitative analysis, presented in Table 2, demonstrate that our method achieved the highest performance in all metrics, with improvements of 3.3% in mIoU, 6.2% in the F1 score, and 6.6% in mAP compared to the best results. Furthermore, visualizations of selected examples in Figure 8 illustrate that our method’s advantages are even more pronounced on unaligned datasets such as FLIR ADAS, producing fewer errors than other methods. These visual results corroborate the quantitative findings, further validating the superiority and effectiveness of our approach.

**Figure 9:**
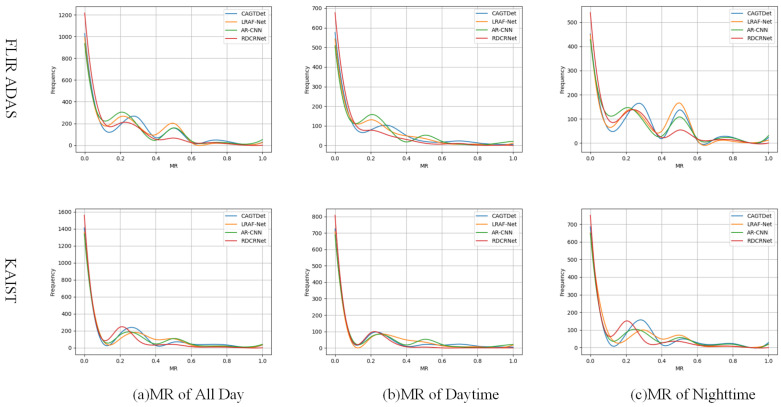
MR values of daytime and nighttime samples for different methods across two datasets.

**Detail of the Result.** To further analyze the detection performance, additional data analysis was performed. A total of 2000 test images were randomly selected from the KAIST and FLIR datasets, with 1000 images each from daytime and nighttime scenes. The miss rate (MR) of various methods under these conditions is presented in Figure 9. In daytime scenes, nearly all cross-modal detection methods exhibited low MR values on both datasets, as the high quality of RGB images in daylight provides abundant target information. In contrast, night-time MR values were higher due to the diminished information from RGB images, although cross-modal methods still outperformed single-modal approaches. RDCRNet achieved the lowest MR values in both scenarios, with less performance degradation at night, indicating its ability to efficiently extract and utilize the limited RGB features available in low-light conditions.

Furthermore, the detection performance for primary categories in the FLIR dataset was analyzed, as shown in Table 3. Pedestrian and vehicle classes, which appear frequently and benefit from strong complementary RGB-T features, were detected more accurately by RDCRNet. This can be attributed to the robust information interaction and aggregation capabilities of our network architecture. For other categories with fewer training samples, all methods demonstrated reduced effectiveness. However, RDCRNet, with its cross-modal pretraining, adapted better to small-sample data, demonstrating superior robustness across diverse object categories.

**Figure 10:**
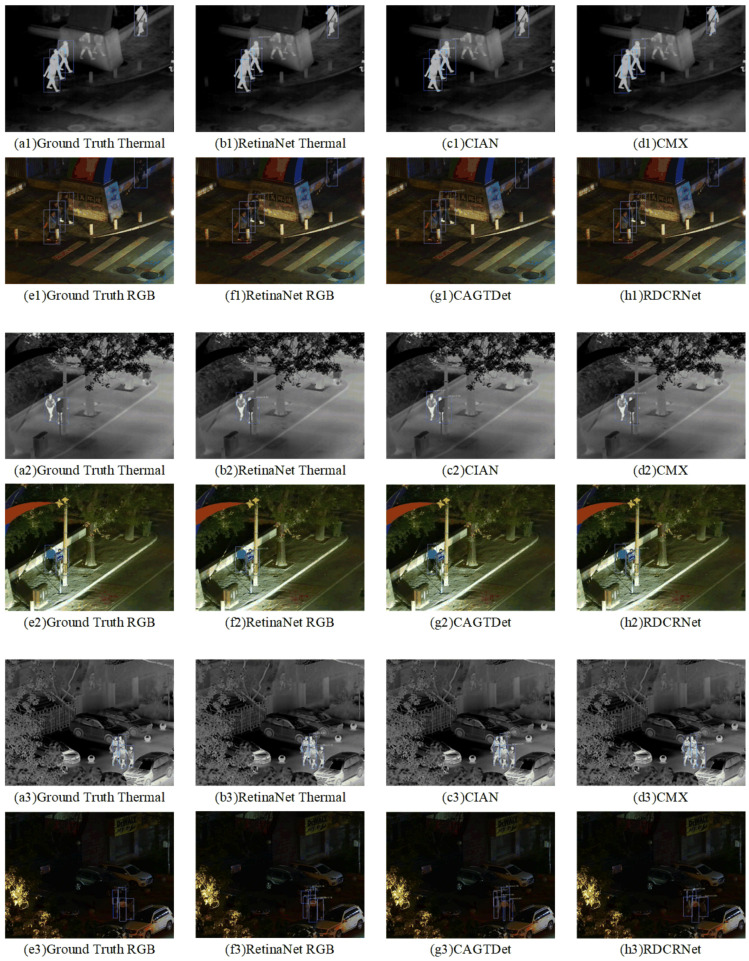
Qualitative comparison of various algorithms under the cross-domain dataset (LLVIP).

**Cross-Domain Dataset.** Experiments conducted on the surveillance scene dataset (LLVIP) further validate the cross-domain generalization capability of RCDRNet. Compared to the ADAS dataset, the LLVIP dataset presents more complex scenes, diverse environmental features, and significant variations in lighting conditions, such as low light, strong light, and shadows, thereby mitigating potential data biases present in road scene datasets. The experimental results demonstrate that RCDRNet exhibits remarkable performance advantages in object detection tasks within surveillance contexts, maintaining high accuracy and robustness under both complex backgrounds and varying lighting conditions. Quantitative analysis, as shown in Table 4, indicates that RCDRNet outperforms the second-best method, achieving an improvement of 3.5% in mIoU, 4.6% in F1 score, and 4.0% in mAP. Qualitative analysis illustrated in Figure 10 showcases selected detection results across different scenarios, revealing that RCDRNet achieves higher detection rates and precision. These experiments indicate that RCDRNet demonstrates strong adaptability to diverse scenes, with performance gains not limited to the specific characteristics of the ADAS domain, but, rather, stemming from the model’s superior architectural design and generalization capabilities. This finding further substantiates the broad applicability of RCDRNet in cross-domain tasks and its potential for deployment in various real-world applications.

### 4.3. Ablation Study

To validate the effectiveness and robustness of key modules in the proposed RDCRNet, several ablation experiments were conducted. For consistency, all ablation experiments followed identical training and testing procedures.

**Figure 11:**
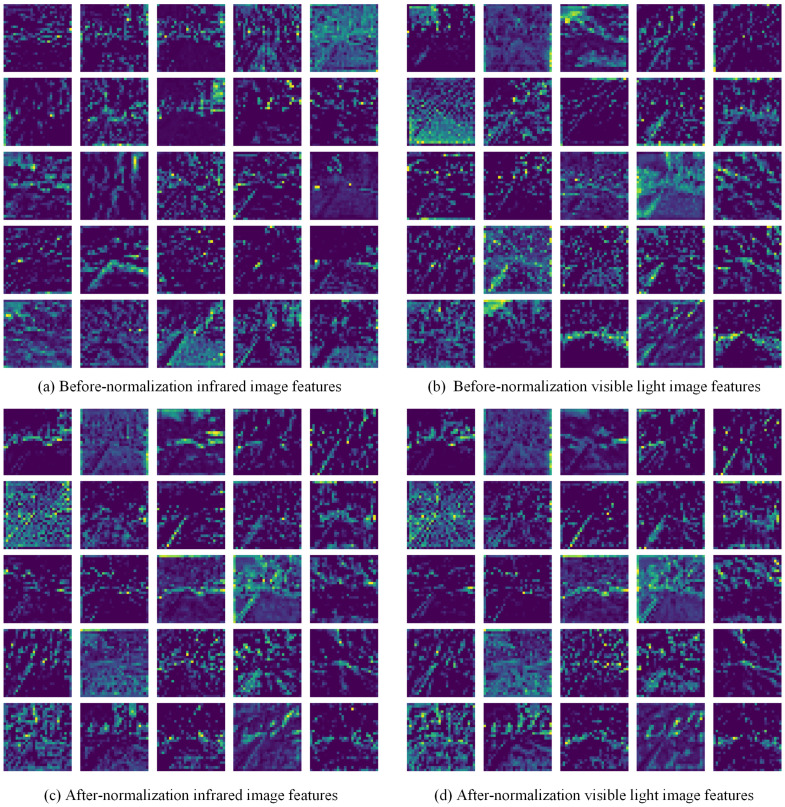
Comparison of infrared and visible light image features before and after normalization.

**Effectiveness of CFR.** To determine if the CFR module effectively facilitates cross-modal feature remapping and enhances network performance, three experimental setups were designed: without remapping, with layer normalization only, and with complete CFR integration. As shown in Table 5, the addition of layer normalization resulted in limited performance improvement, indicating that normalization alone cannot fully address the modality discrepancies. However, when the complete CFR module was introduced, significant performance gains were observed. Visualization of features before and after remapping, illustrated in Figure 11, demonstrates that CFR effectively reduces the distinct differences between infrared and visible features, mapping them to a similar feature space. These results underscore the importance of the CFR module, as it effectively enables the model to harness the complementary capabilities of RGB-T information.

**Figure 12:**
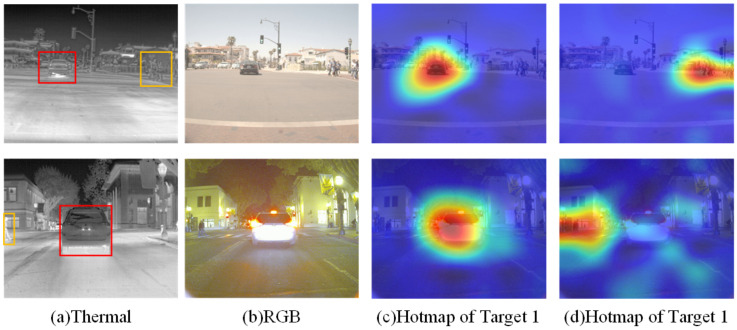
The visualization of semantic feature matching results in CAM.

**Effectiveness of CRIM.** To test the design and necessity of the CRIM module, an ablation study was conducted on various combinations of TRM, CIM, and Feed-Forward Networks (FFNs) within the CRIM module. The results, as shown in Table 6, demonstrate that TRM, by establishing extensive contextual relationships within a single modality, yields moderate performance improvement. However, CIM serves as the core component for cross-modal information interaction, with its absence causing notable performance degradation. To visually demonstrate the effect of information interaction, an RGB-T image pair was selected to observe the cross-modal attention responses within the CRIM module in RDCRNet. As shown in Figure 12, by averaging response values across all CRIM layers and displaying them as red and blue heatmaps, it becomes evident that RDCRNet effectively captures complementary semantic information from the RGB images.

**Figure 13:**
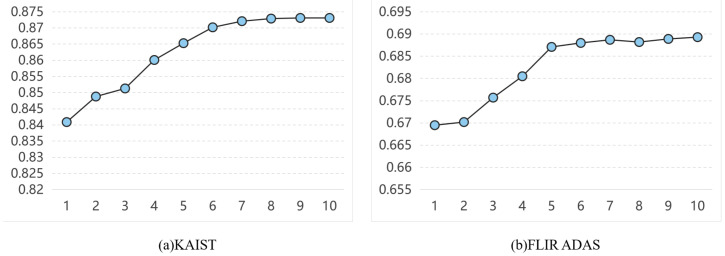
The performance variation curve of detection with respect to the quantity of CRIM and CFI.

**Result for the number of CRIM.** The influence of CRIM layers on network performance was examined to determine optimal hyperparameter settings. As shown in Figure 13, network performance improved gradually with an increase in CRIM layers, but eventually reached a point of diminishing returns. Therefore, to maintain computational efficiency in RDCRNet, N = 6 layers was chosen as the optimal configuration, effectively balancing network complexity and detection accuracy.

**Effectiveness of Cross-Modal Representation Pretraining.** The effectiveness of cross-modal representation pretraining was explored by analyzing four main configurations: no pretraining, no cross-modal reconstruction loss, no semantic consistency loss, and full cross-modal pretraining. The results in Table 7 indicate that the cross-modal pretraining method effectively leverages extensive RGB-T data to acquire prior knowledge, significantly improving detection accuracy. Furthermore, encoder pretraining on the ImageNet dataset was performed for the CRN, which further enhanced the model’s overall performance.

**Figure 14:**
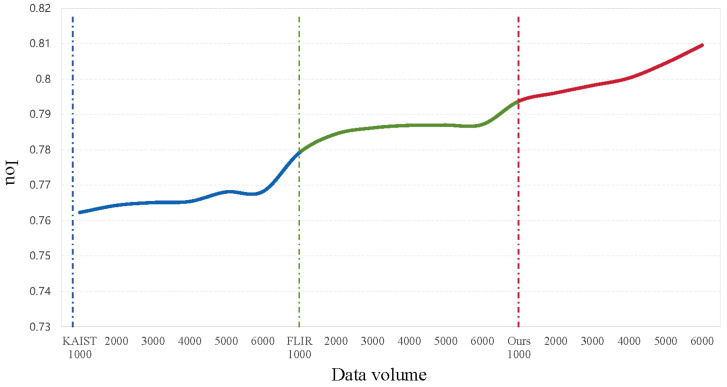
The curve of the effect of the number of unlabeled data on the model performance during pretraining.

**Study of the Dependence of Pretraining Strategies on Unlabeled Data.** To investigate the impact of unlabeled data quantity on model performance during the pretraining process, we designed and conducted a series of ablation experiments. First, we incrementally increased the number of samples within a single dataset; the experimental results are shown in Figure 14. The experiments revealed that model performance improved significantly in the initial stages, particularly after adding approximately 2000 samples, where a substantial enhancement in network performance was observed. However, due to the relatively homogeneous scenes in publicly available datasets (e.g., KAIST and FLIR ADAS), the amount of novel prior knowledge that could be provided diminished gradually. Consequently, when the number of pretraining samples exceeded half the total size of the each dataset, the performance gains began to plateau.

Finally, we conducted a detailed analysis of aligned and non-aligned pretraining samples by categorizing them into three scenarios: (1) only non-aligned samples, (2) only aligned samples, and (3) a combination of aligned and non-aligned samples. To eliminate the influence of sample quantity on the experimental results, we strictly controlled the sample size across the three scenarios, ensuring that they remained at the same order of magnitude. The results, summarized in Table 8, indicate that RDCR-Net achieved the best performance under Scenario 3. This suggests that mixed samples provide richer prior knowledge and effectively enhance the network’s ability to extract and fuse cross-modal information.

**Effectiveness of the CFI Module.** We evaluated the effectiveness of the CFI module by replacing it with two alternative fusion methods: element-wise addition and channel-wise concatenation, while preserving the multiscale feature fusion. Furthermore, we examined the effect of completely removing the multiscale fusion, opting instead for a basic upsampling and fusion strategy similar to the one employed in YOLO. The results, as presented in Table 9, demonstrate that both the CFI module and multiscale feature fusion substantially improve detection performance, confirming that these architectural enhancements are both effective and essential for optimal RGB-T object detection.

## 5. Conclusions

In this paper, we proposed RDCRNet, a novel framework for RGB-T object detection that addresses critical challenges in cross-modal feature alignment, distribution discrepancy mitigation, and efficient fusion under both aligned and unaligned conditions. The core innovation lies in the Cross-Modal Representation Model, which integrates several key components: the Cross-Modal Feature Remapping Module, for statistical normalization and adaptive correction of modality-specific distributions; the Cross-Modal Refinement and Interaction Module, enabling bidirectional feature fusion through trinity refinement and cross-attention mechanisms; and the Cross-Scale Feature Integration Module, for multiscale detection capability enhancement. To overcome data scarcity in RGB-T tasks, we introduced a self-supervised pretraining strategy combining masked reconstruction with adversarial learning and semantic consistency constraints, effectively leveraging both aligned and unaligned multimodal data. Extensive experiments on KAIST, FLIR ADAS, and LLVIP datasets demonstrated state-of-the-art performance, with RDCRNet achieving significant improvements in mIoU, F1 score, and mAP over existing methods while maintaining computational efficiency. The framework’s robustness was particularly evident in challenging low-light scenarios and cross-domain applications, validating its ability to exploit complementary RGB-T information through effective cross-modal representation learning. These advancements establish RDCRNet as a practical solution for real-world applications requiring reliable object detection under diverse environmental conditions.

## Figures and Tables

**Table 1:** Quantitative analysis results of various algorithms under the registered dataset (KAIST). The best and second-best values are highlighted in bold red and blue, respectively.

Method	Modality	mIoU	Recall	Precision	F1 score	mAP	FLOPS (G)	Param (M)
Faster R-CNN	RGB	0.7239	0.7962	0.7675	0.7816	0.6947	16.0	41.0
RetinaNet	RGB	0.7453	0.8325	0.7948	0.8132	0.7337	24.0	35.0
Faster R-CNN	Thermal	0.7520	0.8392	0.8046	0.8216	0.7422	16.0	41.0
RetinaNet	Thermal	0.7515	0.8396	0.8050	0.8219	0.7464	24.0	35.0
IAF R-CNN	RGB-T	0.8013	0.8753	0.8332	0.8537	0.8405	30.0	50.0
Halfway Fusion	RGB-T	0.8040	0.8775	0.8503	0.8637	0.8441	28.0	48.0
CIAN	RGB-T	0.8065	0.8837	0.8597	0.8715	0.8472	70.36	55.0
AR-CNN	RGB-T	0.8109	0.8892	0.8696	0.8793	0.8573	66.0	53.0
LRAF-Net	RGB-T	0.8085	0.8889	0.8693	0.8790	0.8529	40.5	**18.8**
CMX	RGB-T	0.8122	0.8895	0.8753	0.8834	0.8611	**104.3**	28.7
CAGTDet	RGB-T	**0.8248**	**0.8998**	**0.8800**	**0.8898**	**0.8655**	58.60	35.0
RDCRNet(Ours)	RGB-T	**0.8688**	**0.9302**	**0.8992**	**0.9046**	**0.8962**	**101.7**	**16.4**

**Table 2:** Quantitative analysis results of various algorithms under the unaligned dataset (FLIR ADAS). The best and second-best values are highlighted in bold red and blue, respectively.

Method	Modality	mIoU	Recall	Precision	F1 score	mAP
Faster R-CNN	Thermal	0.6931	0.7409	0.7644	0.7524	0.5664
RetinaNet	Thermal	0.6960	0.7312	0.7603	0.7455	0.5732
FCOS	Thermal	0.7105	0.7478	0.7824	0.7647	0.5952
EfficientDet	Thermal	0.7099	0.7439	0.7927	0.7675	0.6098
Cascade-DETR	Thermal	0.7150	0.7723	0.8134	0.7923	0.6491
AR-CNN	RGB-T	0.7397	0.8039	0.8403	0.8217	0.6655
LRAF-Net	RGB-T	0.7406	0.8487	0.8645	0.8262	0.6823
CAGTDet	RGB-T	**0.7840**	**0.8824**	**0.8937**	**0.8378**	**0.7195**
RDCRNet	RGB-T	**0.8096**	**0.9119**	**0.9241**	**0.8897**	**0.7671**

**Table 3:** Detection accuracy of various algorithms for different categories of targets under the registration dataset (KAIST). The specific number of each category is indicated after the category name. The best and second-best values are highlighted in red and blue bold, respectively.

Method	People (5779)	Bicycles (471)	Cars (5432)	Dogs (14)	Total
AR-CNN	**0.6813**	0.3227	0.6794	0.2857	0.6655
LRAF-Net	0.6791	0.2929	0.6771	**0.4285**	0.6623
CAGTDet	0.6810	**0.4140**	**0.6802**	0.3571	**0.6695**
RDCRNet	**0.6860**	**0.7430**	**0.6830**	**0.8571**	**0.6871**

**Table 4:** Quantitative analysis results of various algorithms under the cross-domain dataset (LLVIP). The best and second-best values are highlighted in bold red and blue, respectively.

Method	Modality	mIoU	Recall	Precision	F1 score	mAP
Faster R-CNN	Thermal	0.6712	0.7234	0.7456	0.7343	0.5893
RetinaNet	Thermal	0.6853	0.7351	0.7582	0.7465	0.5967
FCOS	Thermal	0.6968	0.7513	0.7771	0.7633	0.6056
EfficientDet	Thermal	0.6897	0.7442	0.7834	0.7661	0.6102
Cascade-DETR	Thermal	0.7056	0.7601	0.8024	0.7816	0.6234
AR-CNN	RGB-T	0.7256	0.7943	0.8311	0.8122	0.6411
LRAF-Net	RGB-T	0.7348	0.8297	0.8591	0.8341	0.6583
CMX	RGB-T	0.7542	0.8563	0.8722	0.8286	0.6741
CAGTDet	RGB-T	**0.7732**	**0.8745**	**0.8926**	**0.8361**	**0.7257**
RDCRNet	RGB-T	**0.8012**	**0.8971**	**0.9152**	**0.8763**	**0.7556**

**Table 5:** Ablation study results of CFR.

Method	KAIST		FLIR ADAS	
	mIoU	mAP	mIoU	mAP
Without remapping	0.8012	0.8503	0.7418	0.6617
Layer normalization	0.8025	0.8581	0.7426	0.6682
CFR	0.8688	0.8962	0.8096	0.7671

**Table 6:** Ablation study results of CRIM.

Method			KAIST		FLIR ADAS	
FFN	TRM	CIM	mIoU	mAP	mIoU	mAP
×	×	×	0.7631	0.7567	0.7025	0.5944
✓	×	×	0.7652	0.7588	0.7198	0.6458
✓	✓	×	0.8098	0.8119	0.7839	0.7118
✓	✓	✓	0.8688	0.8962	0.8096	0.7671

**Table 7:** Ablation study results of Cross-Modal Representation Pretraining.

Pretraining Step			KAIST		FLIR ADAS	
Classification	CCL	CMAE	Precision	mAP	Precision	mAP
×	×	×	0.7787	0.7982	0.7528	0.6675
✓	×	×	0.8003	0.8013	0.7543	0.6708
✓	✓	×	0.8251	0.8301	0.7889	0.6725
✓	✓	✓	0.8688	0.8692	0.8096	0.7671

**Table 8:** Ablation experiment results on aligned and unaligned pretraining samples.

Method	KAIST		FLIR	
	mIoU	mAP	mIoU	mAP
scenarios 1	0.8234	0.8755	0.7789	0.6920
scenarios 2	0.8215	0.8740	0.7766	0.6943
scenarios 3	0.8688	0.8962	0.8096	0.7671

**Table 9:** Ablation study results of CFI.

Method	KAIST		FLIR	
	mIoU	mAP	mIoU	mAP
w/o Multiscale	0.8023	0.8526	0.7601	0.6754
Add	0.8113	0.8667	0.7678	0.6818
Concat	0.8103	0.8652	0.7655	0.6831
CFI	0.8688	0.8962	0.8096	0.7671

## Data Availability

The original contributions presented in this study are included in the article. Further inquiries can be directed to the corresponding author.

## References

[1] Chen K., Liu J., Zhang H. (2023). IGT: Illumination-guided RGB-T object detection with transformers. Knowl.-Based Syst..

[2] Zhang J., Zhang R., Yuan W., Liu Y. (2024). Rgb-t semantic segmentation based on cross-operational fusion attention in autonomous driving scenario. Evol. Syst..

[3] Wu Y., Guan X., Zhao B., Ni L., Huang M. (2023). Vehicle detection based on adaptive multi-modal feature fusion and cross-modal vehicle index using RGB-T images. IEEE J. Sel. Top. Appl. Earth Obs. Remote. Sens..

[4] Li G., Wang Y., Liu Z., Zhang X., Zeng D. (2022). RGB-T semantic segmentation with location, activation, and sharpening. IEEE Trans. Circuits Syst. Video Technol..

[5] Jiang N., Wang K., Peng X., Yu X., Wang Q., Xing J., Li G., Guo G., Ye Q., Jiao J. (2021). Anti-uav: A large-scale benchmark for vision-based UAV tracking. IEEE Trans. Multimed..

[6] Wang G., Jiang Q., Jin X., Wozniak M., Yao P., Wang S. (2025). RTM-UAVDet: A Real-Time Multimodal UAV Detector. IEEE Trans. Aerosp. Electron. Syst..

[7] Zhu D., Zhan W., Jiang Y., Xu X., Guo R. (2022). IPLF: A novel image pair learning fusion network for infrared and visible image. IEEE Sens. J..

[8] Zhang X., Demiris Y. (2023). Visible and infrared image fusion using deep learning. IEEE Trans. Pattern Anal. Mach. Intell..

[9] Zhang Z., Wang J., Han Y. Saliency prototype for rgb-d and rgb-t salient object detection. Proceedings of the 31st ACM International Conference on Multimedia.

[10] Jiang Y., Liu Y., Zhan W., Tang Y., Li J., Liu Y. (2024). Cross-modal texture transformer for thermal infrared reference-based super-resolution reconstruction. Opt. Laser Technol..

[11] Zhou W., Gong T., Lei J., Yu L. (2023). DBCNet: Dynamic bilateral cross-fusion network for RGB-T urban scene understanding in intelligent vehicles. IEEE Trans. Syst. Man Cybern. Syst..

[12] Ha Q., Watanabe K., Karasawa T., Ushiku Y., Harada T. MFNet: Towards real-time semantic segmentation for autonomous vehicles with multi-spectral scenes. Proceedings of the 2017 IEEE/RSJ International Conference on Intelligent Robots and Systems (IROS).

[13] Zhou W., Liu J., Lei J., Yu L., Hwang J.-N. (2021). GMNet: Graded-feature multilabel-learning network for RGB-thermal urban scene semantic segmentation. IEEE Trans. Image Process..

[14] Zhang J., Liu H., Yang K., Hu X., Liu R., Stiefelhagen R. (2023). CMX: Cross-modal fusion for RGB-X semantic segmentation with transformers. IEEE Trans. Intell. Transp. Syst..

[15] Feng D., Haase-Schütz C., Rosenbaum L., Hertlein H., Glaeser C., Timm F., Wiesbeck W., Dietmayer K. (2020). Deep multi-modal object detection and semantic segmentation for autonomous driving: Datasets, methods, and challenges. IEEE Trans. Intell. Transp. Syst..

[16] Zhang Q., Huang N., Yao L., Zhang D., Shan C., Han J. (2019). RGB-T salient object detection via fusing multi-level CNN features. IEEE Trans. Image Process..

[17] Wang J., Song K., Bao Y., Huang L., Yan Y. (2021). CGFNet: Cross-guided fusion network for RGB-T salient object detection. IEEE Trans. Circuits Syst. Video Technol..

[18] Wang H., Song K., Huang L., Wen H., Yan Y. (2023). Thermal images-aware guided early fusion network for cross-illumination RGB-T salient object detection. Eng. Appl. Artif. Intell..

[19] Wang K., Lin D., Li C., Tu Z., Luo B. (2024). Alignment-Free RGBT Salient Object Detection: Semantics-guided Asymmetric Correlation Network and A Unified Benchmark. IEEE Trans. Multimed..

[20] Tian C., Zhou Z., Huang Y., Li G., He Z. (2024). Cross-Modality Proposal-guided Feature Mining for unaligned RGB-Thermal Pedestrian Detection. IEEE Trans. Multimed..

[21] Chen G., Shao F., Chai X., Chen H., Jiang Q., Meng X., Ho Y.-S. (2022). Modality-induced transfer-fusion network for RGB-D and RGB-T salient object detection. IEEE Trans. Circuits Syst. Video Technol..

[22] Kyrkou C., Theocharides T. (2011). A parallel hardware architecture for real-time object detection with support vector machines. IEEE Trans. Comput..

[23] Kyrkou C., Theocharides T. (2010). A flexible parallel hardware architecture for AdaBoost-based real-time object detection. IEEE Trans. Very Large Scale Integr. (VLSI) Syst..

[24] Li Z., Liu F., Yang W., Peng S., Zhou J. (2021). A survey of convolutional neural networks: Analysis, applications, and prospects. IEEE Trans. Neural Networks Learn. Syst..

[25] Girshick R., Donahue J., Darrell T., Malik J. Rich feature hierarchies for accurate object detection and semantic segmentation. Proceedings of the IEEE Conference on Computer Vision and Pattern Recognition.

[26] Girshick R. Fast R-CNN. Proceedings of the IEEE International Conference on Computer Vision.

[27] Ren S., He K., Girshick R., Sun J. (2016). Faster R-CNN: Towards real-time object detection with region proposal networks. IEEE Trans. Pattern Anal. Mach. Intell..

[28] Xie X., Cheng G., Wang J., Yao X., Han J. Oriented R-CNN for object detection. Proceedings of the IEEE/CVF International Conference on Computer Vision.

[29] Sun P., Zhang R., Jiang Y., Kong T., Xu C., Zhan W., Tomizuka M., Yuan Z., Luo P. (2023). Sparse r-cnn: An end-to-end framework for object detection. IEEE Trans. Pattern Anal. Mach. Intell..

[30] Redmon J. You only look once: Unified, real-time object detection. Proceedings of the IEEE Conference on Computer Vision and Pattern Recognition.

[31] Redmon J., Farhadi A. YOLO9000: Better, faster, stronger. Proceedings of the IEEE Conference on Computer Vision and Pattern Recognition.

[32] Farhadi A., Redmon J. (2018). Yolov3: An incremental improvement. Comput. Vis. Pattern Recognit..

[33] Varghese R., Sambath M. YOLOv8: A novel object detection algorithm with enhanced performance and robustness. Proceedings of the 2024 International Conference on Advances in Data Engineering and Intelligent Computing Systems (ADICS).

[34] Liu W., Anguelov D., Erhan D., Szegedy C., Reed S., Fu C.-Y., Berg A.C. SSD: Single shot multibox detector. Proceedings of the Computer Vision–ECCV 2016: 14th European Conference.

[35] Tian Z., Shen C., Chen H., He T. (2020). FCOS: A simple and strong anchor-free object detector. IEEE Trans. Pattern Anal. Mach. Intell..

[36] Tan M., Pang R., Le Q.V. EfficientDet: Scalable and efficient object detection. Proceedings of the IEEE/CVF Conference on Computer Vision and Pattern Recognition.

[37] Ye M., Ke L., Li S., Tai Y.-W., Tang C.-K., Danelljan M., Yu F. Cascade-DETR: Delving into high-quality universal object detection. Proceedings of the IEEE/CVF International Conference on Computer Vision.

[38] He K., Chen X., Xie S., Li Y., Dollár P., Girshick R. Masked autoencoders are scalable vision learners. Proceedings of the IEEE/CVF Conference on Computer Vision and Pattern Recognition.

[39] Bachmann R., Mizrahi D., Atanov A., Zamir A. (2022). Multimae: Multi-modal multi-task masked autoencoders. Eur. Conf. Comput. Vis..

[40] Tao M., Bao B.-K., Tang H., Xu C. Galip: Generative adversarial clips for text-to-image synthesis. Proceedings of the IEEE/CVF Conference on Computer Vision and Pattern Recognition.

[41] Sun X., Wang Z., Lu Z., Lu Z. (2024). Self-supervised graph representations with generative adversarial learning. Neurocomputing.

[42] Radford A., Kim J.W., Hallacy C., Ramesh A., Goh G., Agarwal S., Sastry G., Askell A., Mishkin P., Clark J. Learning transferable visual models from natural language supervision. Proceedings of the International Conference on Machine Learning.

[43] Li J., Li D., Xiong C., Hoi S. Blip: Bootstrapping language-image pretraining for unified vision-language understanding and generation. Proceedings of the International Conference on Machine Learning.

[44] Hwang S., Park J., Kim N., Choi Y., So Kweon I. Multispectral pedestrian detection: Benchmark dataset and baseline. Proceedings of the IEEE Conference on Computer Vision and Pattern Recognition.

[45] Li C., Song D., Tong R., Tang M. (2019). Illumination-aware Faster R-CNN for robust multispectral pedestrian detection. Pattern Recognit..

[46] Liu J., Zhang S., Wang S., Metaxas D.N. Multispectral deep neural networks for pedestrian detection. Proceedings of the IEEE Conference on Computer Vision and Pattern Recognition.

[47] Zhang L., Liu Z., Zhang S., Yang X., Qiao H., Huang K., Hussain A. (2019). Cross-modality interactive attention network for multispectral pedestrian detection. Inf. Fusion.

[48] Zhang L., Zhu X., Chen X., Yang X., Lei Z., Liu Z. Weakly aligned cross-modal learning for multispectral pedestrian detection. Proceedings of the IEEE/CVF International Conference on Computer Vision.

[49] Fu H., Wang S., Duan P., Xiao C., Dian R., Li S., Li Z. (2023). LRAF-Net: Long-range attention fusion network for visible–infrared object detection. IEEE Trans. Neural Networks Learn. Syst..

[50] Yuan M., Shi X., Wang N., Wang Y., Wei X. (2024). Improving RGB-infrared object detection with cascade alignment-guided transformer. Inf. Fusion.

